# Diagnosis of retinal disorders from Optical Coherence Tomography images using CNN

**DOI:** 10.1371/journal.pone.0254180

**Published:** 2021-07-27

**Authors:** Nithya Rajagopalan, Venkateswaran N., Alex Noel Josephraj, Srithaladevi E.

**Affiliations:** 1 Department of Biomedical Engineering, Sri Sivasubramaniya Nadar College of Engineering, Chennai, India; 2 Department of Electronics and Communication Engineering, Sri Sivasubramaniya Nadar College of Engineering, Chennai, India; 3 Department of Electronic Engineering, College of Engineering, Shantou University, Shantou, China; Vellore Institute of Technology, INDIA

## Abstract

An efficient automatic decision support system for detection of retinal disorders is important and is the need of the hour. Optical Coherence Tomography (OCT) is the current imaging modality for the early detection of retinal disorders non-invasively. In this work, a Convolution Neural Network (CNN) model is proposed to classify three types of retinal disorders namely: Choroidal neovascularization (CNV), Drusen macular degeneration (DMD) and Diabetic macular edema (DME). The hyperparameters of the model like batch size, number of epochs, dropout rate, and the type of optimizer are tuned using random search optimization method for better performance to classify different retinal disorders. The proposed architecture provides an accuracy of 97.01%, sensitivity of 93.43%, and 98.07% specificity and it outperformed other existing models, when compared. The proposed model can be used for the large-scale screening of retinal disorders effectively.

## 1. Introduction

The eye is the light of human life. The light enters the eye through the cornea, passing through the aqueous humor, lens, vitreous humor, and finally on to the retina lying on the back of eye [1]. The retina is the most important part of the eye. It is divided into pigment epithelium, receptor layer, cell layer, receptor layer, internal limiting membrane, external limiting membrane, and vitreous body. The retina’s photoreceptor cells use the external light that the cornea focuses through the lens and convert it into nerve signals, transported to the brain through the optic nerve.

According to WHO’s Blindness and vision impairment statistics in 2019, around 2.2 billion people suffer from blindness or vision impairment, out of which 80% of disability can be avoided if detected at an early stage [1, 2]. Around 6.9 million people have glaucoma, 3 million people have diabetic retinopathy, and 2.75 million people have AMD and Duren. Also, WHO (2019) estimates that more than 360 million people will be affected worldwide by diabetes mellitus by 2030. All these people will be at risk of developing diabetic macular edema [3].

With such massive numbers, detecting retinal disorders manually by ophthalmologist is a strenuous task. The identification of retinal disorders can be made by spotting the existence of deformation associated with the disease. Although clinicians may be able to diagnose the disease through vascular abnormalities, its resource demands are high. In underdeveloped countries where the diabetic population is high, this equipment may not be readily accessible. The major challenges are mass screening, skilled technician, observer variability and, early detection. An automated alternative is more efficient, reliable, and the need of the hour. The main contribution of the work is to develop an algorithm for detecting retinal disorders towards building an efficient decision support system.

Diabetic Macular Edema (DME) causes retinal inflammation and leakage of blood vessels due to Diabetic Retinopathy (DR) [4]. The prevalence of DME in DR subjects is 2.7% - 11%. Common factors affecting DME occurrence are ethnicity, gender, proteinuria, cardiovascular diseases, and Diabetes. In Drusen Macular Degeneration (DMD), the occurrence of yellow or white deposits between the sub retinal layers were more common. Risk of macular degeneration is high with the progression of such deposits leading to vision loss [5–7]. In Choroid Neovascularization (CNV) condition, the non-vascularized blood vessel enters the RPE cells and causes vascular leakage. In CNV, it is observed that new blood vessels emerge from the choroidal region, which often causes hemorrhage. The advanced stage of CNV results in the thickening of the Retinal Pigment Epithelium (RPE) layer of the retina. Detecting retinal disorders at an early stage is used to prevent disease progression and vision loss [8].

An ophthalmologist uses two major imaging modalities, namely color fundus imaging and OCT. The 2D image of the retina is well represented in the color fundus image. The fundus camera captures the light reflected from the retina and forms the fundus image. In a color fundus image, deformation in the retina can be identified. But the depth information degeneration cannot be accessed. The OCT is a non-invasive imaging modality mainly used in ophthalmology to visualize retinal layers [9, 10]. Information about all the retina layers can be inferred from OCT images, useful in detecting and diagnosing retinal disorders [8, 10]. Early detection of retinal disorders can be done effectively using OCT compared to fundus photography. Even a minimal change in retinal layers can be accurately seen in the OCT images, as it acquires the cross-sectional view from the sub retinal layers. Fundus photography can be used only for visualizing the 2-dimensional view of the retina and lacks providing depth information about the retinal layer [11, 12]. The interferometric technique’s properties are defined by the signal sampling at the detector and the light source’s coherence properties. With this unique OCT property, the retina’s high-resolution image is achieved. Timed domain OCT and Frequency domain OCT are different type of acquisition domains. The light source used in TD-OCT is usually a super luminescent diode and reference beam length is varied. Frequency domain OCT (FD-OCT) uses separate detectors to acquire the broadband interference.

In this work the author develops a CNN architecture for detection of retinal disorders using OCT images. The rest of the paper is organized as follows. Related work is discussed in section II. The results and discussion for the proposed architecture is provided in section IV. Section V subsumes the conclusion and course of action for the future.

## 2. Related works

Retinal disorders are detected and diagnosed by performing retinal layer segmentation and thickness measurement in the retinal OCT image. The retinal layers’ changes due to any disorder were not common, and fixing a specific benchmark process is impossible for analyzing the data. Standard image processing algorithms for retinal layer abnormality detection have some difficulties, such as time-consuming, sufficient domain knowledge. Also, a generalization of the process for automatic processing is difficult [13, 14]. A convolution neural network is a recent tool that involves image classification, image recognition, and image retrieval [15–17].

Often-occurring retinal complications, which involve damage to the optic disc, macular region, rods, cones, and blood vessels, supply and nourish the retina, resulting in vision loss. For the detection and treatment of retinal disorders, machine learning algorithms have been widely employed. Machine learning algorithms yield a function that can accurately predict class labels based on a training algorithm. The retinal image is processed through a sequential process of image pre-processing, segmentation, feature extraction, supervised and unsupervised classification methods to detect various retinal disorders [18]. For automated DR detection, [19] proposed a transfer learning-based CNN on binocular retinal fundus images. A hybrid deep learning model to detect retinal lesions automatically is presented [20]. A CNN algorithm for DR grading. With less volume of dataset, the performance decreased during testing [21].

CNN can effectively extract features and classify retinal OCT images [22, 23]. Rare retinal disorders like inferior staphyloma, chorioretinal atrophy, Vogt—Koyanagi—Harada (VKH) the disease can also be effectively detected using the CNN model [24]. Several CNN networks are framed for various applications. The LeNet network has few free parameters, and also, the network could be trained on a low-level representation of data that has minimal pre-processing [25]. Another network called AlexNet has improved performance over LeNet architecture. AlexNet was the first deep network architecture used for Image classification to classify a thousand classes [26, 27]. This network is used for the DR detection using retinal fundus images. Three stages of DR have been classified with an average accuracy of 96% [28]. The VGG-16 architecture has been proposed for obtaining high-level features [29, 30] and the number of parameters used in the architecture is 138 million parameters. The complexity of VGG-16 is high compared to the Alexnet [31]. With the CNN model, image classification [32] and disease diagnosis can be made efficiently with less processing time [17, 33, 34]. Most of the literature concentrates on fundus image for retinal disorder detection which gives only two-dimensional information [18, 20, 21]. The OCT image is able to detect the disease at an early stage, with the available three-dimensional information of the retinal layers. Recently researchers have concentrated on OCT images. Noise removal and disease diagnosis is a challenging task in OCT images [14, 35, 36]. A simple network with high efficiency is developed to detect retinal disorders using OCT images.

Analysis on noise removal is implemented to remove the speckle noise in the OCT images for improving the efficiency of the system. Development of a simple CNN model for four class classification of retinal disorders and tuning its hyperparameters using random search optimization method. In most of the CNN models, a transfer learning approach is used that modifies the existing architecture for necessary applications to achieve better efficiency. In this paper, a novel CNN model is proposed having less complexity, low computational time and tuned hyperparameters. Also, in this work a four-class classification is performed for retinal disorders detection.

**Fig 1:**
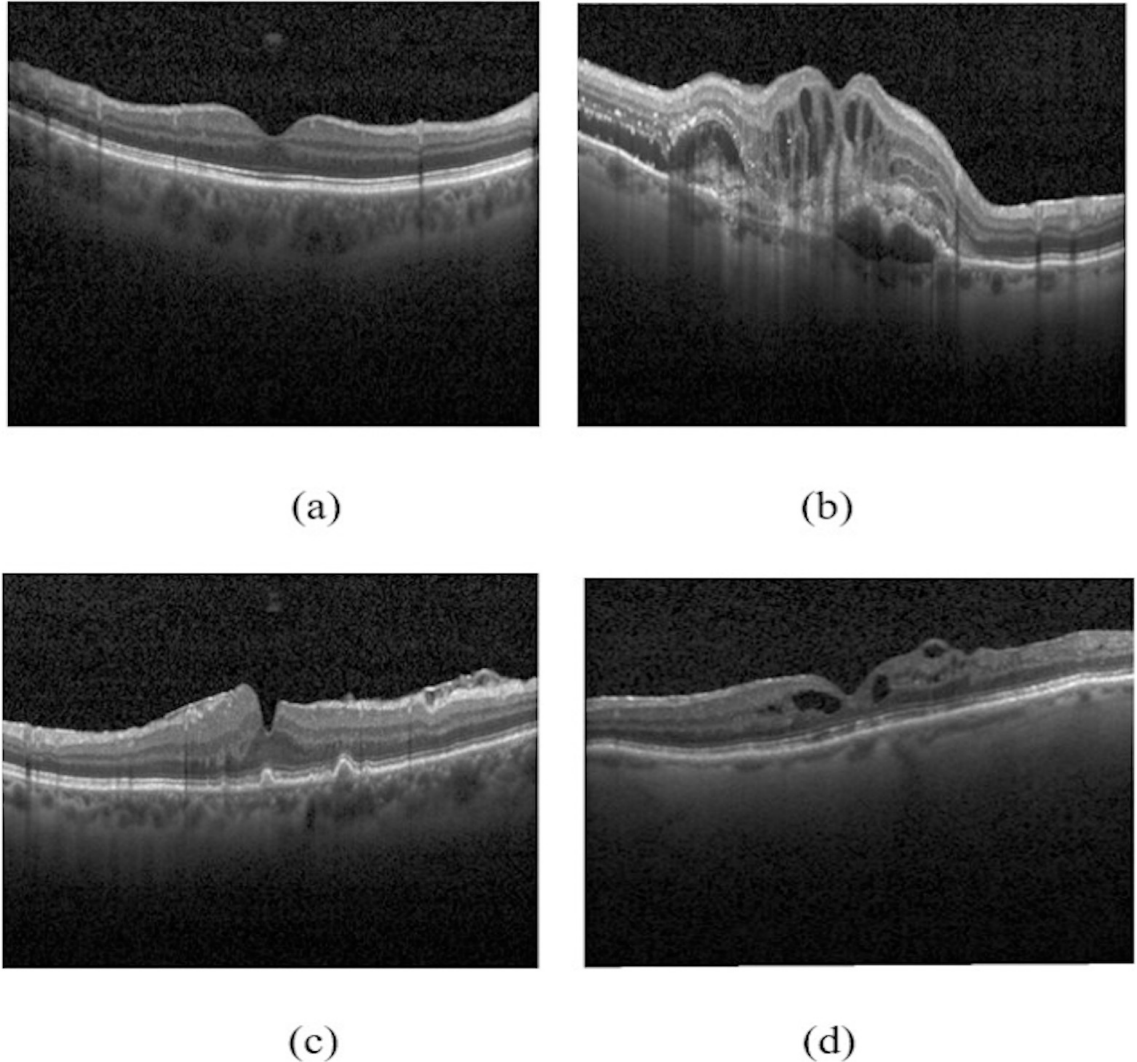
Retinal OCT images of (a) Normal (b) CNV (c) DMD (d) DME.

This research aims to classify retinal disorders using a novel CNN model with higher accuracy than the existing model. The features of convolution layers are also visualized. The OCT retinal images of normal and abnormal conditions are shown in Fig 1(A)–1(D).

The Mendeley database consists of around 84,000 OCT images are categorized into four types namely Diabetic Macular Edema, Drusen Macular Degeneration, Choroidal Neovascularization and Normal [35]. The retinal OCT of CNV, DME, DMD and normal images were used in this study. From the database, 12000 images have been used in the classification process, 3000 images from each category.

## 3. Pre-processing of OCT images

Pre-processing the images is to improve the quality of the image so that interpretation without error is possible, and enhance some image features that are important for further processing. Generally, OCT images have speckle noise, a granular noise commonly found in medical images. It is a multiplicative expressed as shown in Eq 1.


c(i,j)=o(i,j)*m(m,n)+a(m,n) (1)

Where *c*(*i*,*j*) is noisy image, *o*(*i*,*j*) original image, *u*(*i*,*j*) is the multiplicative parameter, and *η*(*i*,*j*) is the additive parameter.

This type of speckle noise reduction improves the visual perception and system accuracy during classification. The Retinal OCT images are to be pre-processed for removing speckle noise. Firstly, the images are resized to equal dimensions since they are all of the different sizes. Secondly, in the speckle noise reduction process, different filters are used to compare performance factors. Both spatial domain–convolving the noisy image with mask, and frequency domain filters–transforming the noisy image to frequency domain and apply filters are used for speckle-noise reduction. The spatial filter acts on an image by reducing the intensity variation between adjacent pixels. The simple sliding-window spatial filter replaces itself and pixels that are unrepresentative of their surroundings. It is implemented with a convolution square mask that provides a result which is a weighted sum of the values of a pixel and its neighbors. If the mask’s sum coefficients are to unit value, then the average brightness of the image is not changed, however while the sum gives zero, the average brightness is lost, and it returns a dark image. The common speckle filters such as Kuan, mean filter, biorthogonal spline wavelet, and wiener filter are considered for this study.

Kuan filter is an additive smoothening filter that changes the multiplicative speckle parameter into an additive linear parameter. It is often used to remove speckle noise from the radar and medical images using minimum mean square error calculation to estimate the signal’s value for the center cell in the window. It then calculates the signal estimate from the variance, local mean, and standard deviation. The weighted function is expressed as in Eq 2.


$$W=\frac{1-(\frac{C_{u}^{2}}{{c_{i}}^{2}})}{1+(C_{u}^{\;2})}$$ (2)

Where *Cu* is the estimated noise variation coefficient, and it is expressed as: *Cu* = $\sqrt{1/ENL}$, *ENL* is equivalent noise looks.

*Ci* = *S*/*m* - Variation coefficient of image

*S* = Standard deviation in the filter window, m = mean with in the filter window

Mean filters are simple and intuitive filters which reduce the amount of variation in intensity between one pixel and the next. Mean filter simply replaces each pixel value in an image with its neighbors’ mean value, including itself. By eliminating pixel values that are unrepresentative of surroundings, the speckle noise is suppressed. It computes the sum of all pixels in the sliding window and then divides the sum by the number of pixels in the filter window. The result gives a blurring effect with some loss of details along with reduced speckle noise.

For a*b window region, a mathematical representation of the mean filter is given as in the Eq 3.


$$h(i,j)=\frac{1}{ab}\sum_{k\in a}\;\sum_{l\in b}\;f(m,n)$$ (3)

The biorthogonal wavelets introduced by Cohen-Daubechies-Feauveau (CDF) wavelets are arguably the second most popular family of wavelets in image processing. A variant of these with four primal and dual vanishing moments is used in the JPEG2000 standard. They are also popular in finite element computations in scientific computing because the elementary scaling functions can be B-spline. This means they are piecewise polynomials, and they can be represented exactly with analytical expressions as in Eq 4.


H(k)(π)=0,k=0,...,p−1andH˜(k)(π)=0,k=0,...,q−1 (4)

The Wiener filter is a linear spatial domain filter. It can restore corrupted or blurred images. It can also be implemented in both spatial domain (mean squared method for denoising) and frequency domain (Fourier transforms for denoising and blurring operation). It works on the basis of computation of specific statistical parameters apart from usual parameters (mean, Variance), both the locally (higher order moments of the kernel) and globally (higher-order moment of the entire image), and the statistical properties in the image differ from one region to another. The larger the local variance, the lesser the smoothing effect, and if the local variance is small. It is represented mathematically as in Eq 5.


$$f(u,v)=[\frac{H{\left(u,v\right)}^{*}}{H{\left(u,v\right)}^{2}+[\frac{sn(u,v)}{sf(u,v)}]}]w(u,v)$$ (5)

Where,

*H*(*u*, *v*)^2^ = Degradation function and

*H*(*u*, *v*)* = Conjugate complex

*w*(*u*, *v*) = Degraded image

*sn*(*u*, *v*) = Power spectra of noise

*sf*(*u*, *v*) = Power spectra of original image

To analyze above mentioned filtering techniques three parameters namely, Peak Signal-to-Noise Ratio (PSNR), Mean Square Error (MSE), and structural similarity index (SSIM) are calculated. The noisy image and filtered image are considered for performance analysis of the filter. When the PSNR values are low, with high MSE and SSIM value, the filter performance is high.

Mean Square Error (MSE) is the cumulative squared error between the filtered and the original image it expressed as in Eq 6.


$$MSE=\frac{{\sum_{A,B}\;I_{1}(i,j)-I_{2}(i,j)]}^{2}}{A*B}$$ (6)

Where,

*I*_1_(*i*,*j*) = Original image

*I*_2_(*i*,*j*) = Approximated version of the image (filtered image) and *A*,*B* = Dimensions of the images

Peak Signal-to-Noise Ratio (PSNR) is the ratio between the maximum possible power and the power of noise. It is expressed as in Eq 7.


$$PSNR=20\log_{10}(\frac{{\left(N-1\right)}^{2}}{MSE})\;\text{db},$$ (7)

Where N = representing the number of gray levels. Structural similarity Index (SSIM) is a perceptual metric that measures image quantity degradation caused by compression due to processing or loss in data transmission. It is also a full reference metric requiring both the original image and the measurement’s processed image. SSIM can be defined as in Eq 8.


$$SSIM=\frac{1}{M}\sum \;\frac{\left(2\mu_{1}\mu_{2}+C_{1})(2\sigma_{1,2}+C_{2}\right)}{\left({\mu_{1}}^{2}+{\mu_{2}}^{2})({\sigma_{1}}^{2}+{\sigma_{2}}^{2}+C_{2}\right)}$$ (8)

Where μ1, μ2 - the mean value of the original and the filtered image

*σ*_1_, *σ*_2_ - the standard deviations of the original and the filtered image.

*σ*_1,2_- the covariance between the original and the filtered image.

**Fig 2:**
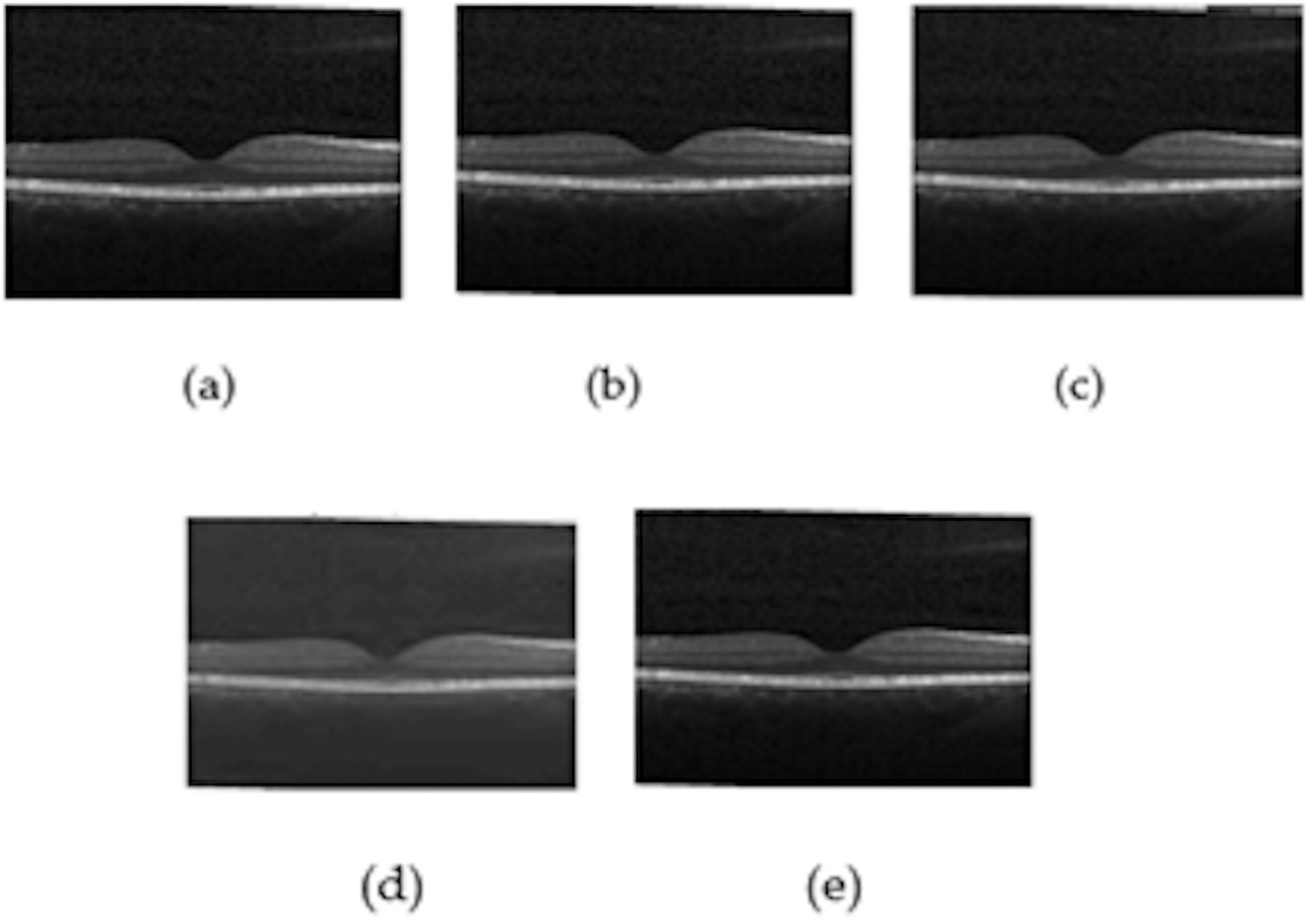
Output of the Speckle Reduction Filters (a) Original image, (b) Biorthogonal Spline wavelet (c) Mean filter, (d) Kuan filter, and (e) Wiener filter.

The parameter value varies between 0 and 1, and low value represents structurally dissimilar. The images in the dataset are of different sizes. Therefore, all the images are resized to equal size to have an equal number of pixels. Four different filters are used for speckle noise reduction. The filters are, mean filter, wiener filter, Kuan and biorthogonal spline wavelet filter. For analyzing the speckle reduction, the quality assessment metrics are calculated. Original and filtered images are considered for the measurement. The PSNR, MSE, and SSIM of the various filters used are represented in the Table 1, from that it is inferred biorthogonal spline wavelet based filter outperformed the other three. Fig 2 shows the output of filters applied on the OCT images. The input images were preprocessed and then fed to the proposed CNN Model for classification.

**Table 1:** Performance analysis of noise removal.

Filter	Image class	PSNR (dB)	MSE	SSIM	Computation time (sec)
**Biorthogonal Spline Wavelet**	**Normal**	**5.30**	**191.89**	**0.26**	**1.14**
	**DME**	**5.25**	**154.00**	**0.15**	
	**DMD**	**5.90**	**167.13**	**0.14**	
	**CNV**	**5.64**	**177.36**	**0.22**	
Kuan filter	Normal	5.27	193.04	0.28	36.41
	DME	5.16	197.87	0.14	
	DMD	6.04	161.83	0.24	
	CNV	5.85	168.72	0.28	
Mean filter	Normal	5.28	192.43	0.26	1.86
	DME	6.12	158.61	0.13	
	DMD	5.73	173.67	0.19	
	CNV	5.59	179.32	0.26	
Wiener filter	Normal	5.22	165.17	0.29	0.83
	DME	6.17	157.01	0.15	
	DMD	6.77	122.00	0.23	
	CNV	5.55	131.16	0.29	

## 4. Convolution neural network

Some basic components required for framing CNN are convolution layers (CL), pooling layers (PL), and a fully connected layer (FCL). The convolution layer uses the activation functions like ReLU, softmax etc., followed by the pooling layer. This pattern was repeated until the image is merged spatially to reduce its size. Following this, it was connected to a fully connected layer (FCL). The output can be obtained from FCL. These layers were stacked to form the full CNN architecture. The CNN components are described below. The CL specification can be defined by parameters that include filter size, strides, padding, and spatial size (width, height, and depth). Each filter in the layer slides over the input volume’s spatial size and calculates the dot product between the filter and input volume elements. The results obtained were passed into the non-linear activation function. ReLU is the activation function followed by each CL. ReLU function is the most commonly used activation, which works much faster than other activation functions like tanh or sigmoidal function [32]. The PL is inserted in between CL in the architecture. The PL’s function is to make a progressive reduction in the size of the input and for the computation of parameters. The fully connected layer (FCL) is the last layer of the convolution architecture. The function of FCL is to connect all neurons from the former layer to the single neuron layer. The softmax activation function is the most commonly used in the final layer of CNN because of its better probability distribution.

### 4.1 Proposed CNN architecture

The proposed CNN Architecture contains five Convolution Layers (CL) and two fully connected Layers (FL). The CNN components were described below. The CL specification can be defined by parameters that include filter size, strides, padding, and spatial size (width, height, and depth). Each filter in the layer slides over the input volume’s spatial size and calculates the dot product between the filter and input volume elements. The results obtained were passed into the non-linear activation function. ReLU is the activation function followed by each CL. The PL is inserted in between CL in the architecture. The PL’s function is to make a progressive reduction in the size of the input and computation of parameters. The fully connected layer (FCL) is the last layer of the convolution architecture. The function of FCL is to connect all neurons from the former layer to the single neuron layer. The original images were in different dimensions. The images were resized into uniform size to enable extraction of the features. Filtered for speckle noise reduction. The input image was fed into the network with a spatial dimension of 224x224x1. For this work, 12,000 retinal greyscale images were considered, 3,000 images in each of four categories. The dataset was split for training and testing. Using the K- fold method, the images were split into 8000 images for training and 4,000 for testing. The validation split was 0.1 from the training dataset. Less complexity and hyperparameter optimization are the advantage of the proposed architecture. The computation time to train the network takes 4s for each epoch. The model is implemented with a 32 core AMD processor, 64GB RAM with NVidia 2060RTX series GPU.

The resized images were passed through the stack of CLs. The ReLU activation function was used in the CLs, which converged faster than other activation functions. The max-pooling layer was followed by the CLs (not all convolution layers) with a common pool size of (2,2) with strides of 2. Two FLCs followed the CLs. The output layer was the softmax activation layer, which classified the input into four classes. The stochastic gradient descent (SGD) optimizer was used to reduce the error rate and metrics like accuracy and loss function. Overfitting the model can be avoided by choosing the optimal dropout rate [24, 37].

**Fig 3:**
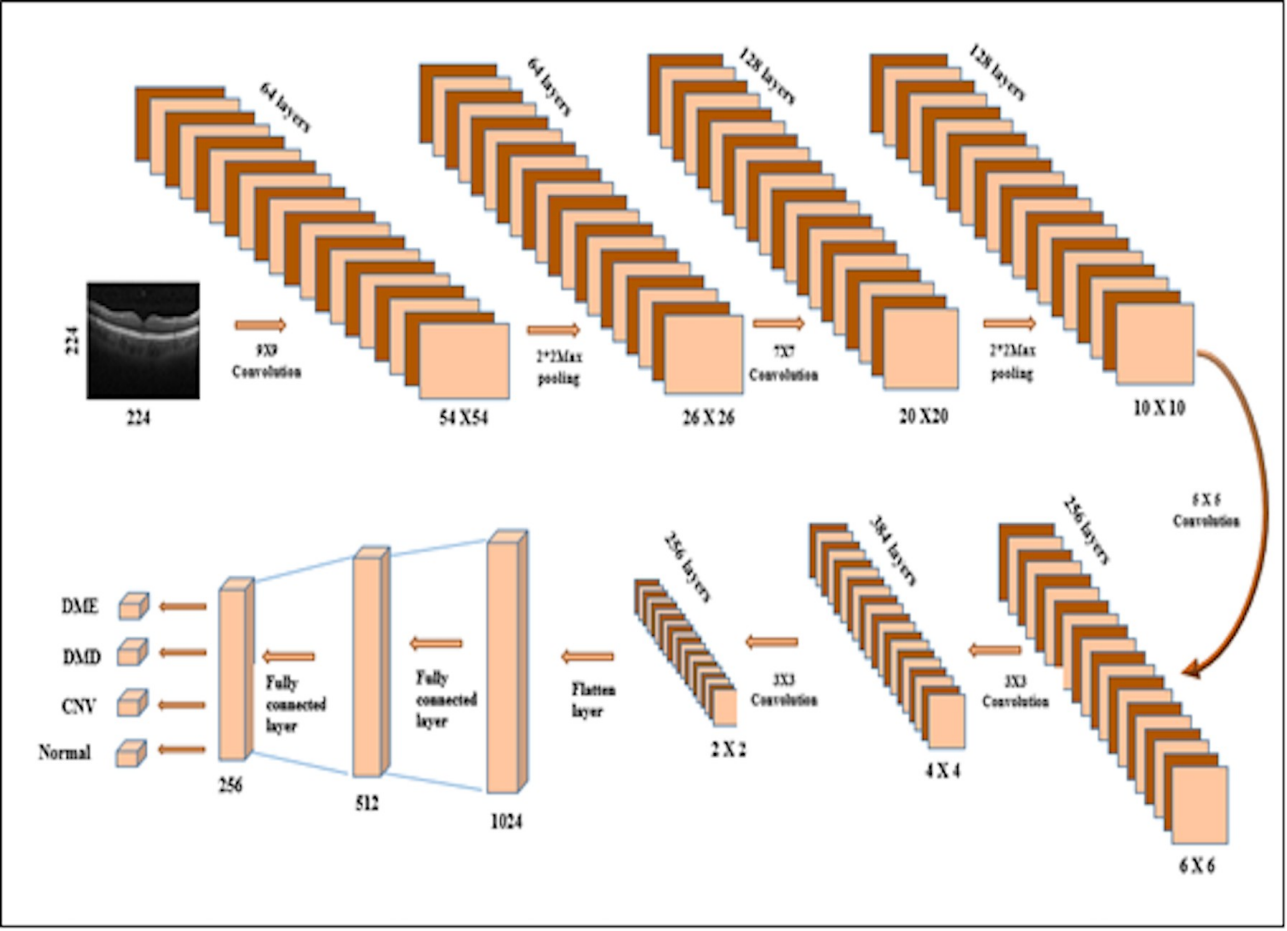
Proposed CNN architecture.

Batch Normalization is a method commonly used in CNNs to normalize a set of inputs to the layer. The input layer can be normalized by adjusting and scaling the activations. It has several advantages, namely, its ability to reduce overfitting, activation adjustment, and its mutual relationship with the dropout layer. In the proposed architecture, the batch normalization is incorporated in the Convolutional and Dense layers. The proposed network architecture is shown in Fig 3.

The output size and trainable parameters of proposed CNN layers were computed using Eqs 9 and 10.


$$\mathrm{Output}\;\mathrm{size}=\frac{\mathrm{Image}\;\mathrm{size–Filter}\;\mathrm{size}}{\mathrm{Stride}+1}$$ (9)

Parameters=((Kernelsize×Depthslice)+Bias)×Numberoffilter (10)

The details about the layers in the proposed CNN model are given in Table 2.

**Table 2:** Parameters of proposed CNN architecture.

Layers	Name	No. of filters	Kernel size	Stride	Output size	Trainable parameters
0	Input	-	-	-	-	0
1	Convolution	64	9 x 9	4	54 x54x64	5248
2	Max- pooling	64	2 x 2	2	26x26x64	0
3	Convolution	128	7 x 7	1	20x20x128	401536
4	Max- pooling	128	2 x 2	2	10x10x128	0
5	Convolution	256	5 x 5	1	6x6x256	819456
6	Convolution	384	3 x 3	1	4x4x384	885120
7	Convolution	256	3 x 3	1	2x2x256	884992
8	Flatten layer	-	-	-	1024	0
9	Dense layer	-	-	-	512	524800
10	Dense layer	-	-	-	256	131328
11	Output layer	-	-	-	4	1028

### 4.2 Hyperparameters optimization

The critical task of deep learning is to choose the best hyperparameters for the model. The hyperparameter selection by manual search involves many attempts and costs serious. So, the authors moved on to optimize the hyperparameters. The hyperparameters used in deep learning were learning rate, batch size, epochs, and optimizer. There are several approaches for hyperparameter tuning. The most widely used hyperparameter tuning technique is the random search. Random search is a technique that selects a combination of hyperparameters for training the model [38]. In this investigation, a random search was performed. The hyperparameters were tuned for batch size, epochs, dropout rate, and optimizer. The hyperparameters were evaluated using mean and standard deviation. The optimized hyperparameters are shown in Table 3.

**Table 3:** Optimized hyperparameters.

Hyperparameters	Parameters	Optimized parameter
Batch size	10,20,40,60,80,100	100
Epochs	10,50,100	50
Dropout rate	0.1,0.2,0.3,0.4,0.5	0.2
Optimizer	SGD, rmsprop, adam	SGD

### 4.3 Feature visualization

**Fig 4:**
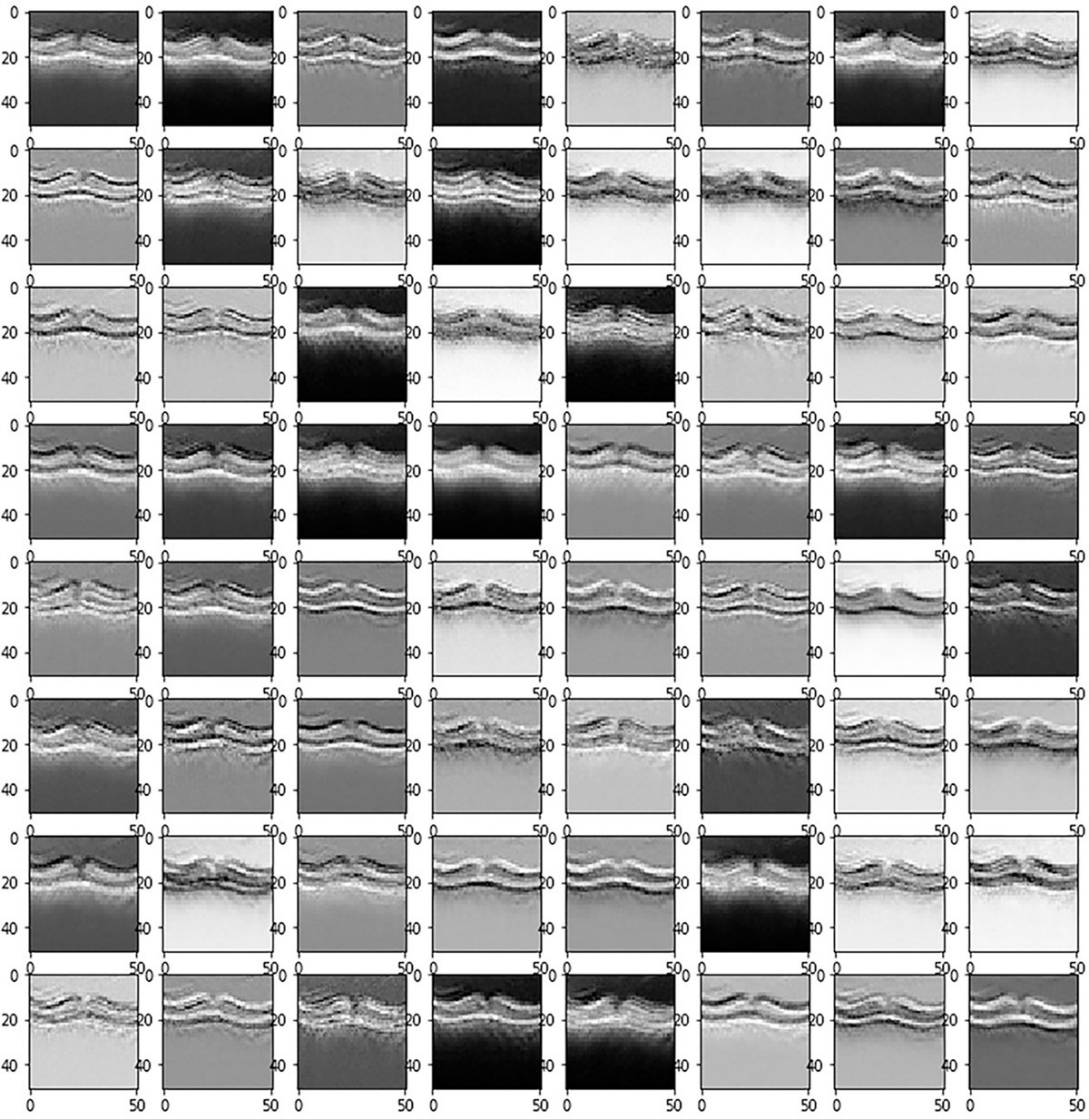
Visualization of features of first convolution layers.

The performance of each convolution layer can be seen by visualizing the output of each layer. Feature visualization explicitly shows the functionality of each CNN layer [39]. Initial layers extract the edge information by neighbor comparison and advanced layers extract higher level features used for classification. Fig 4 visualizations output of the first convolution layer (64 filters), highlighting the edge information.

## 5. Experimental results and discussion

**Fig 5:**
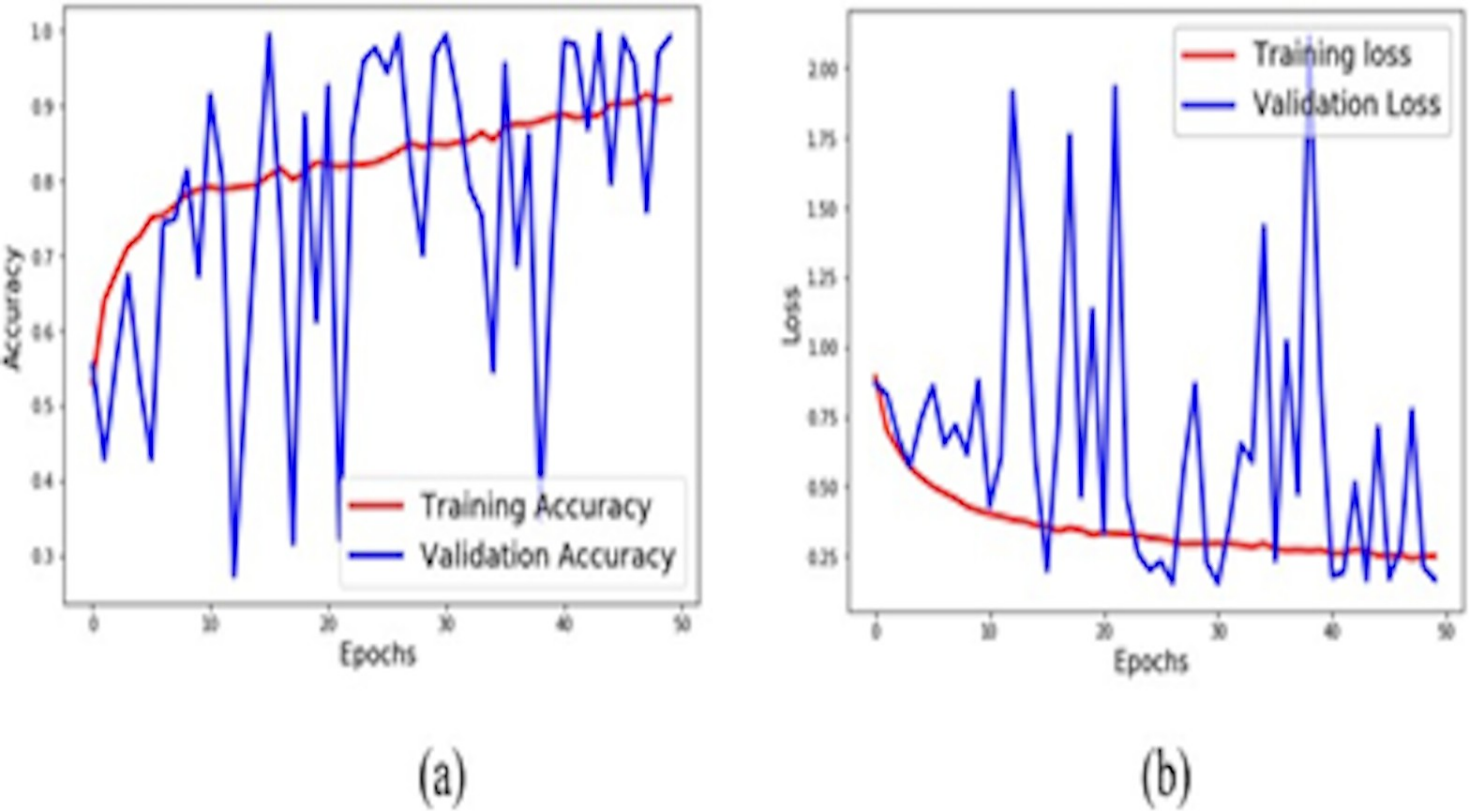
(a) Accuracy curve (b) Loss curve.

The network is trained with 8000 images, where 2000 were trained in each of the four classes. The proposed network architecture’s accuracy and loss curve is shown in Fig 5(A) & 5(B). The validation split took 0.1% of the data from the four classes. The proposed network achieved validation accuracy was 98.4%, whereas the training loss was reduced to 25% and validation loss to 10%.

### 5.1 Choice of K value

K-fold cross-validation helps improve the efficiency of the customized model. The k-fold divides the data into k groups and gets trained by each data. Therefore, it predicts easily when the model explores unseen data with the same features as the trained images. In this work, the k values set were assigned to 3, 4, and 5. This provided the ability to measure the proposed model’s performance to achieve a higher testing accuracy with three folds. With the increase in k value, the accuracy decreases, as shown in Table 4.

**Table 4:** Performance measure of K fold validation.

Details	K-Fold split		
	K = 3	K = 4	K = 5
Number of images for Training	8000	9000	9600
Number of images for Testing	4000	3000	2400
Average Testing Accuracy (%)	97.01	89.3	77.04

The above result shows the parameters like average testing accuracy of the proposed model reaching a higher accuracy for threefold.

### 5.2 Confusion matrix for four classes (3-Fold)

The customized model performed a multi-class classification. The network was tested with 4000 images, with 1000 drawn from each of the four categories. The classifier distinguished between urgent referrals like CNV, DME, and DMD from normal.

Table 5 shows that the classifier was correctly predicting 961 images as CNV, 923 images DME, 926 images as DMD, and 933 images as normal. Prediction of the remaining cells in the confusion table was erroneous. Performance metrics were calculated from the confusion matrix and tabulated in Table 6.

**Table 5:** Confusion matrix.

N = 4000		Predicted			
		CNV	DME	DMD	NORMAL
**Actual**	**CNV**	961	16	18	5
	**DME**	16	923	26	35
	**DMD**	12	8	926	54
	**NORMAL**	2	24	41	933

**Table 6:** Performance metrics.

Label	Sensitivity	Specificity	Accuracy
**CNV**	96.10	99.00	98.37
**DME**	92.30	98.40	96.88
**DMD**	92.00	98.00	96.92
**Normal**	93.30	96.87	95.88
Average accuracy			97.01

### 5.3 Performance comparison

The proposed convolution neural network was compared with existing architectures. The dataset was trained and tested with LeNet and AlexNet. The architectural complexity is less in these two networks similar to the developed model. So, these models were trained and tested with the same dataset. The performance of the proposed model is compared and found outperforming as in Table 7.

**Table 7:** Comparison with existing networks.

Networks	Testing accuracy (%)	Sensitivity (%)	Specificity (%)
AlexNet	87.7	90.48	85.06
LeNet	52.4	80.7	73.68
**Proposed network**	97.01	93.43	98.07

**Fig 6:**
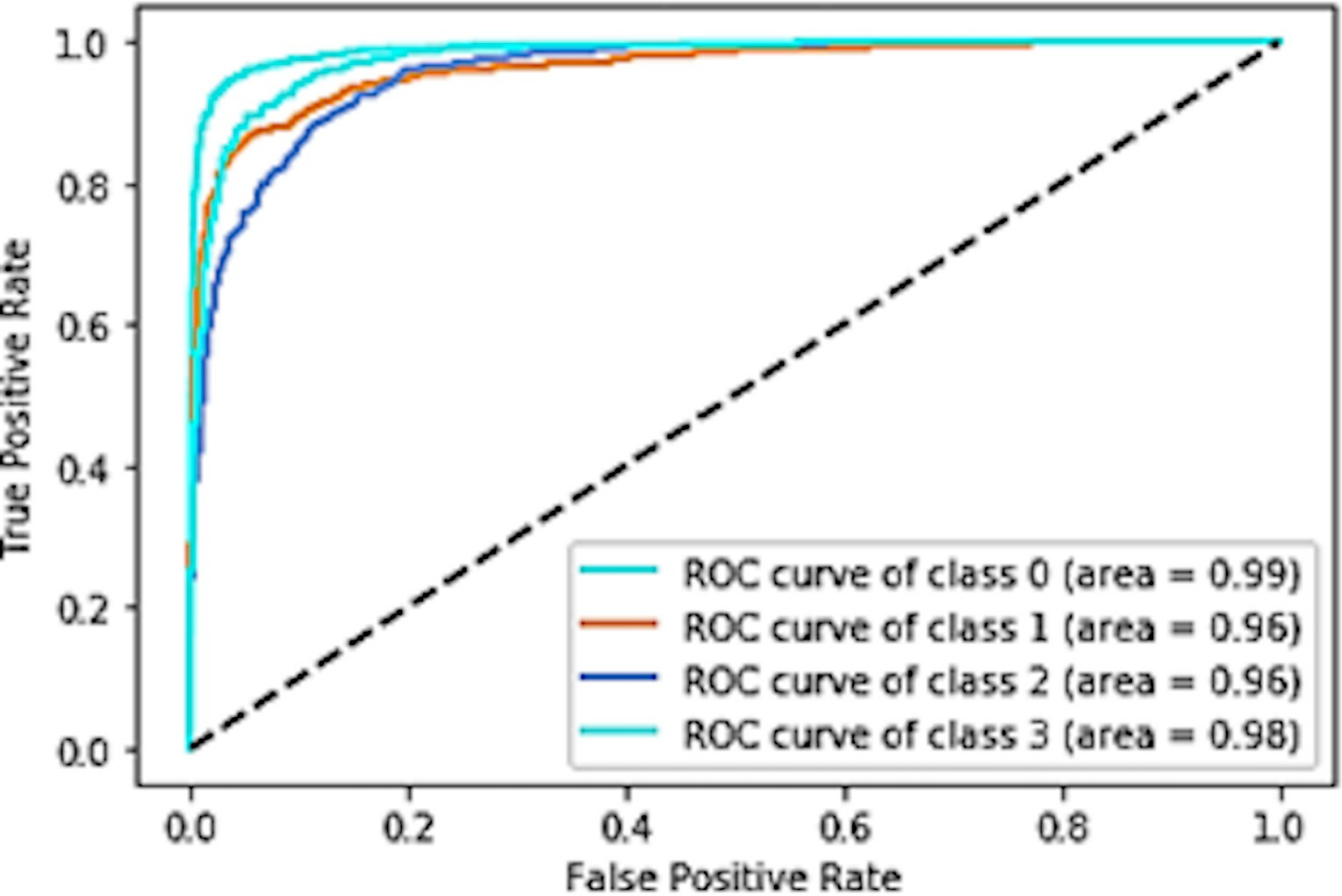
ROC curve for the proposed network architecture.

The receiver operating characteristic (ROC) curve is a graphical plot that shows the classifier’s diagnostic ability. The performance of the classifier was better when the value was higher. If the value is higher, then the performance of the classifier is better. The ROC curve plots the true positive rate (TPR) against the false positive rate (FPR) at different thresholds. The ROC curve of the proposed architecture is shown in Fig 6.

**Fig 7:**
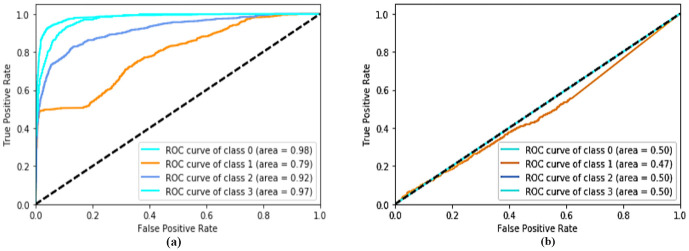
ROC curve for (a) the AlexNet, (b) the LeNet.

The ROC rate for class 0 is 99%, ROC rate for class 1 is 96%, ROC rate for class 2 is 96%, and ROC rate for class 3 is 98%. The ROC curve of AlexNet and LeNet architecture, as shown in Fig 7(A) & 7(B), were obtained and used for comparison with of ROC curve of the proposed network architecture.

The database provider [35] used transfer learning techniques in ImgeNet and achieved an accuracy of 93.4% with a sensitivity and specificity of 96.6% and 94% respectively. The area under the ROC curve classifies the disorders from normal is 98.8%. The accuracy of the proposed model when compared to the above model is better.

The results obtained were compared with the existing research papers and presented in Table 8.

**Table 8:** Comparison with existing work.

Reference	Class	Number of CNN Layers	Accuracy (%)
**Perdomo et al.,** [23]	Normal/DME	12	93.75
**Lee et al.** [40]	Normal /AMD	21	93.45
**Asaoka et al.** [41]	Normal/Glaucoma	4	92.6
**Jie Wang et al.** [42]	Normal/Glaucoma	-	81.4
**Kermany et al.** [35] **(2018) (Mendeley dataset)**	Normal CNV/DMD/DME	9	96.6
**Proposed Model (Mendeley dataset)**	Normal CNV/DMD/DME	12	97.01

The OCT image denoising analysis is performed and found Biorthogonal wavelet transform is better in denoising the image. The denoised image is fed to the proposed CNN model for classification. The results show the proposed model has higher efficiency in the classification of OCT images into disease categories such as CNV, DMD, DME, and Normal. The performance of the network is explained by highlighting the confusion matrix, calculating the average accuracy, sensitivity and specificity. The results of the proposed model are compared with few of the literature and existing models.

## 6. Conclusion

A four-class classification is performed for retinal OCT images using proposed CNN architecture. From the publicly available MENDELY dataset, four classes of images, CNV, DMD, DME, and normal (3000 in each class). The images were denoised using a biorthogonal spline wavelet filter. The denoised images were fed to an eleven-layer CNN architecture framed to classify the retinal disorders. A random search method was used to optimize the hyperparameters. This method can be used for large-scale screening of retinal disorders effectively. The CNN architecture effectively classified urgent referrals like CNV, DME, and DMD from the normal retinal OCT images with a testing accuracy of 97.01%. Effective visualization of the features of the convolution layers is possible. The proposed network has achieved a sensitivity of 93.43% and a specificity of 98.07%. The network proposed in the paper has been optimized for its hyperparameters. The proposed network has been shown to perform better compared to existing models. The proposed techniques’ demonstrated efficacy could help the ophthalmologists in the effective retinal image analysis, thereby providing a better treatment at an early stage, thus preventing blindness. The proposed model is trained and tested only with OCT images of different retinal disorders. The work can be further extended to develop a single flexible CNN architecture that can analyze different modalities of retinal images like ultrasound, fundus images for different disorders. Such a system can be useful in large-scale screening. For analysis, 12000 images are considered, 3000 images in each category CNV, DMD, DME and normal. In future, the dataset size could be increased for enhancing the efficiency of the system. Such a system can be useful in large-scale screening.
